# Rapid identification of allergenic and pathogenic molds in environmental air by an oligonucleotide array

**DOI:** 10.1186/1471-2334-11-91

**Published:** 2011-04-13

**Authors:** Wen-Tsung Hung, Shu-Li Su, Lin-Yi Shiu, Tsung C Chang

**Affiliations:** 1 Environmental Analysis Laboratory, Environmental Protection Administration, Zhongli, Taiwan; 2 Department of Medical Laboratory Science and Biotechnology, College of Medicine, National Cheng Kung University, Tainan, Taiwan

## Abstract

**Background:**

Airborne fungi play an important role in causing allergy and infections in susceptible people. Identification of these fungi, based on morphological characteristics, is time-consuming, expertise-demanding, and could be inaccurate.

**Methods:**

We developed an oligonucleotide array that could accurately identify 21 important airborne fungi (13 genera) that may cause adverse health problems. The method consisted of PCR amplification of the internal transcribed spacer (ITS) regions, hybridization of the PCR products to a panel of oligonucleotide probes immobilized on a nylon membrane, and detection of the hybridization signals with alkaline phosphatase-conjugated antibodies.

**Results:**

A collection of 72 target and 66 nontarget reference strains were analyzed by the array. Both the sensitivity and specificity of the array were 100%, and the detection limit was 10 pg of genomic DNA per assay. Furthermore, 70 fungal isolates recovered from air samples were identified by the array and the identification results were confirmed by sequencing of the ITS and D1/D2 domain of the large-subunit RNA gene. The sensitivity and specificity of the array for identification of the air isolates was 100% (26/26) and 97.7% (43/44), respectively.

**Conclusions:**

Identification of airborne fungi by the array was cheap and accurate. The current array may contribute to decipher the relationship between airborne fungi and adverse health effect.

## Background

Fungi are widely distributed in the natural environment. Fungal spores can be easily dispersed into the air and may cause serious health problems. Exposure to fungal spores can cause a wide spectrum of allergenic reactions, such as asthma, and infections in susceptible individuals [1-4]. Asthma prevalence has considerably increased in recent decades such that it is now one of the most common chronic disorders in the world [5-7]. Some severe diseases, such as allergic bronchopulmonary aspergillosis and fungal sinusitis, may be found in susceptible or immunocompromised individuals through mold exposure [8,9]. The predominant genera of airborne fungi causing health concern are *Alternaria*, *Aspergillus*, *Cladosporium*, and *Penicillium *[4].

In order to decipher the relationship between fungi and potential fungal infection, it is imperative to establish methods that can accurately identify airborne fungi to the species level and the method could be easily followed. Conventional methods for fungal identification are primarily based on morphological and physiological tests [10]. These tests often require several days or even weeks and the results can be inconclusive or inaccurate [11]. Even for a mycologist, the identification of airborne fungi to the species level can be challenging, due to the taxonomically high divergence of these microorganisms. In recent years, numerous DNA-based methods have been developed to identify a variety of medically important fungi [12]. The rRNA genes have been extensively used as the targets for molecular identification [12,13]. These methods include DNA probes [14], PCR-restriction enzyme analysis [15], real-time PCR [16], and DNA sequencing [17,18]. PCR techniques are particularly promising because of their simplicity, sensitivity, and specificity. However, these methods can identify only one or a limited number of species at a time.

A variety of DNA array methods, having the capacity to simultaneously identify multiple targets, have been developed to identify pathogenic fungi [19-23,26,30] with high sensitivity and specificity. In contrast, literatures using the array platform to detect airborne fungi are very limited and so far only one study using nonspecific probes was reported [24]. In our previous studies, oligonucleotide probes designed from the internal transcribed spacer (ITS) regions have been developed to identify a wide variety of pathogenic molds (19,26) and yeasts (30), including some airborne species. The aim of this study was to expand the probe panel to identify 21 airborne fungal species (13 genera) that may cause health problems in susceptible persons.

## Methods

### Fungal strains

A total of 73 target strains (strains we aimed to identify) representing 21 species (13 genera) (Table 1) and 66 nontarget strains (66 species, additional file 1) were used in this study. These strains were obtained from the Bioresources Collection and Research Center (BCRC, Hsinchu, Taiwan), the American Type Culture Collection (ATCC, Manassas, Virginia, USA), and Centraalbureau voor Schimmelcultures (CBS, Utrecht, The Netherlands).

**Table 1:** Fungal strains used for identification by the oligonucleotide array

Species	Strain^a^	Total no. of strains
*Acremoniun strictum*	BCRC 32290, BCRC 32239^T^	2
*Alternaria alternata*	BCRC 32888, BCRC 30501, CBS 105.49	3
*Aspergillus flavus*	BCRC 30006, BCRC 30007, BCRC 30008, BCRC 30009, BCRC 30165^T^	5
*Aspergillus fumigatus*	BCRC 32149, BCRC 30502^T^, BCRC 33373, BCRC 33380, BCRC 33381	5
*Aspergillus niger*	BCRC 320201, BCRC 30204, BCRC 31130^T^, BCRC 30507, BCRC 32720, BCRC 33046	6
*Aspergillus versicolor*	BCRC 31488^T^, BCRC 31123, BCRC 32142, BCRC 30225, BCRC 31895	5
*Aureobasidium pullulans*	BCRC 31981, BCRC 32064, BCRC 32065	3
*Chaetomium cochlioides*	BCRC 31605, BCRC 30523, BCRC 31771	7
*Chaetomium funicola*	CBS 973.73, CBS 378.77	2
*Chaetomium globosum*	CBS 142.88, CBS 766.96	2
*Cladosporium cladosporioides*	BCR 32925, BCRC 30812, BCRC 32887	3
*Mucor racemosus*	BCRC 30186, BCRC 32586, BCRC 30896	3
*Paecilomyces variotii*	BCRC 30562, CBS 112279, CBS 370.70	3
*Penicillium brevicompactum*	BCRC 31258, BCRC 31259, BCRC 33336	3
*Penicillium chrysogenum*	BCRC 30564^T^, BCRC 30563, BCRC 30568, BCRC 30569	4
*Penicillium corylophilum*	BCRC 31555, BCRC 32015 , BCRC 32620	3
*Rhizopus stolonifer*	BCRC 31135, BCRC 31142 , BCRC 31633	3
*Scopulariopsis brevicaulis*	ATCC 7903, ATCC 62614, BCRC 31751	3
*Scopulariopsis chartarum*	CBS 522.69, CBS 897.68, CBS 410.76	3
*Stachybotrys chartarum*	BCRC 32551, BCRC 32552, BCRC 32554, BCRC 32818, BCRC32819, BCRC 32820	6
*Trichoderma viride*	BCRC 32054, BCRC 3312 , BCRC 33458	3
Total strain		77

^a^ATCC, American Type Culture Collection, Manassas, Virginia, USA; BCRC, Bioresources Collection and Research Center, Hsinchu, Taiwan, Republic of China; CBS, Centraalbureau voor Schimmelcultures, Utrecht, The Netherlands.

^T^Type strain.

### DNA extraction

Mycelia (approximately 0.5 × 0.5 cm) grown on Saubouraud dextrose agar were transferred into a 2-ml screw cap tube (Azygen Sientific, Union City, California, USA) containing 300 mg of zirconium/silica beads (0.5 mm in diameter, Biospec Products, Bartlesville, Oklahoma, USA) in 1 ml of sterilized saline. The mycelial suspension was shaken in a cell disrupter (Mini-Beadbeater, Biospec Products) for 5 min at a speed of 4,200 rpm. An 0.1 ml aliquot of the disrupted cell suspension was transferred to a 1.5-ml centrifuge tube and centrifuged at 8,000 × *g *for 10 min. Fungal DNA in the supernatant was extracted by a DNA extraction kit (Viogene, Taipei, Taiwan) following the manufacturer's instructions [19].

### ITS amplification and sequencing

The fungus-specific universal primers ITS1 (5'-TCCGTAGGTGAACCTGCGG-3') and ITS5 (5'-GGAAGTAAAAGTCGTAACAAGG-3') were used as the forward primers [25], while ITS4 (5'-TCCTCCGCTTATTGATATGC-3') was used as the reverse primer to amplify the ITS region [25]. PCR was performed in a total reaction volume of 50 μl consisting of 10 mM Tris-HCl (pH 8.3), 0.08% Nonidet P-40 (Sigma-Aldrich, St. Louis, Minnesota, USA), 50 mM KCl, 1.5 mM MgCl_2_, 0.8 mM deoxynucleoside triphosphates (0.2 mM each), 1.2 U of *Taq *DNA polymerase, 0.7 μM (each) of the forward and reverse primers, and 1 to 5 ng of DNA template. PCR was carried out using the following conditions: initial denaturation at 94°C for 3 min; 35 cycles of denaturation (94°C, 1 min), annealing (60°C, 1 min), and extension (72°C, 1 min); and a final extension step at 72°C for 7 min. PCR products were purified and sequenced by using an ABI Prism 377 automated DNA sequencer (Applied Biosystems, Taipei, Taiwan) with a BigDye Terminator cycle sequencing kit (version 3.1; Applied Biosystems).

### Design of oligonucleotide probes

Species- or group-specific oligonucleotide probes (18- to 30-mers) were designed from the ITS 1 or ITS 2 regions based on sequences in the GenBank database (Table 2) or on sequences determined in this study. The positive control probe was designed from a conserved region in the 5.8S rRNA gene [26]. The designed probes were checked for melting temperature, secondary structure, and GC content by using the Vector NTI Advance 9 (Invitrogen, Carlsbad, California, USA), and checked for potential cross-reactivity with other species in GenBank by using the BLASTN program. A total of 25 probes, including 11 previously described ones [19,26,30], were used to fabricate the oligonucleotide array on nylon membrane. Ten bases of thymine were added to the 3' ends of probes that exhibited weak hybridization signals after preliminary testing [27]. An irrelevant oligonucleotide (16 bases) labelled with a digoxigenin molecule at the 5' end was used as a position marker on the array.

**Table 2:** Oligonucleotide probes used to identify airborne fungi

Microorganism	Probe					
	Code^a^	Sequence (5'-3')^b^	Length (nt)	*T*_*m *_(℃)	Location^c^	GenBank accession no.
*Acremonium strictum*^d^	Acstr2-5	CTGCGTAGTAGCACAACCTCGCAtttttttttt	23	59.1	431-453 (2)	AJ621771
*Alternaria alternata*^e^	Alalt3	CGCACTCTCTATCAGCAAAGGTCTAGCATC	30	63.5	461-490 (2)	AY625056
*Aspergillus flavus*^e^	Asfla4	CGAACGCAAATCAATCTTTTTCCAGGT	27	63.1	512-538 (2)	AY373848
*Aspergillus fumigatus*^e^	Asfum2-1	GCCAGCCGACACCCAACTTTATTTTTCTAAtttttttttt	30	65.4	213-242 (2)	AY230140
*Aspergillus niger*^e^	Asnig2	ACGTTTTCCAACCATTCTTTCCAGGT	26	60.9	517-542 (2)	AY373852
*Aspergillus versicolor*^e^	Asver4	ACGTCTCCAACCATTTTCTTCAGGT	25	58.2	486-510 (2)	AY830119
*Aureobasidium pullulans*^e^	Aupul2	ATTTCTAACAACGCTCTTTGGGTCGGTACG	30	65.5	454-483 (2)	AF121283
*Aureobasidium pullulans*^e^	Aupul3	TCAAAGGAGAGGACTTCTGCCGACTGAAAC	30	66.2	456-485 (2)	AY139395
*Aureobasidium pullulans*^e^	Aupul4	GGCGTAGTAGAATTTATTCGAACGTCTGTC	30	60.7	428-457 (2)	AY139395
*Chaetomium cochliodes/C. globosum/C. funicola*^e^	Chcgf1	GGCCTCTCTGAGTCTTCTGTACTGAATAAG	30	58.8	157-186 (1)	AJ279450
*Cladosporium cladosporioides*	Ccla2-2	CGGGAGGCTACGCCGTAAAtttttttttt	19	57.4	470-488 (2)	AY361994
*Mucor racemosus*	Mrac2-1	GGGCCTCTCGATCTGTATAGATCTTtttttttttt	25	55.3	573-597 (2)	AY625074
*Mucor racemosus*	Mrac3-1	TAGATCTTGAAATCCCTGAAATTTACTtttttttttt	27	52.7	590-616 (2)	AY625074
*Paecilomyces variotii*^f^	Pavar2	CCGAAGACCCCTSGAACGCtttttttttt	19	59.0	155-173 (1)	AY373941
*Penicillium brevicompactum*	Pbre1-1	ACCCGCTTTGTAGGACTGCCCGtttttttttt	22	63.0	439-461 (2)	AY484922
*Penicillium chrysogenum*	Pchr1-1	TCAACCCAAATTTTTATCCAGGtttttttttt	22	52.6	482-503 (2)	DQ674380
*Penicillium corylophilum*	Pcor1-2R	CGCGGGCCAGAGGGCAGAtttttttttttt	18	63.9	102-119 (1)	AF034456
*Penicillium corylophilum*	Pcor2-2R	CGCGGGCCAGAGGGCAGAAGtttttttttttt	20	65.6	100-119 (1)	AF033450
*Rhizopus stolonifer*	Rsto4	AAAGGCGGTTAATGGTATCCAACAAATtttttttttt	27	60.0	246-272 (1)	AB113023
*Scopulariopsis chartarum*	Sccha1	TCTTCATACCCATTTGTGAACACTACCCtttttttttt	28	59.4	41-68 (1)	AY625066
	Sccha4-1	AGTAAAGCACCTCGCATCGGGTCCtttttttttt	24	63.2	497-520 (2)	AY625066
*Scopulariopsis brevicaulis*^e^	Scbre3-1	TGCGTAGTAGATCCTACATCTCGCATCGtttttttttt	28	62.4	500-527 (2)	AY625065
*Stachybotrys chartarum*	Scha1-3	CAGTATTCTCTGAGTGGGAAACGCAAAtttttttttt	27	60.7	485-511 (2)	AF081468
*Stachybotrys chartarum*	Scha1-4	AGTATTCTCTGAGTGGTAAACGCAAAtttttttttt	26	54.8	157-181 (1)	AY095976
*Trichoderma viride*	Tvir2-1	AACCAAACTCTTTCTGTAGTCCCCTCtttttttttt	26	56.5	111-136 (1)	AY380909
Positive control^g^	PC	GCATCGATGAAGAACGCAGCttttttttt	20	55.7	200-219	EF134625

^a^Oligonucleotide probes are arranged on the array as indicated in Figure 1.

^b^Multiple bases of thymine, indicated by "t", were added to the 3' end of the probe. The underlined nucleotide indicates a single mismatch base that was intentionally incorporated into the probe to avoid cross-hybridization.

^c^The location of probe is shown by the nucleotide number of either ITS 1 or ITS 2; the number (1 or 2) in parenthesis indicates the ITS region from which the probe was designed.

^d^Probe modified from a previous study (19)

^e^Probes designed in a previous study (19)

^f^Probe designed in a previous study (26)

^g^The positive control probe was designed from a conserved region of the 5.8S rRNA gene (30)

### Fabrication of arrays

**Figure 1:**
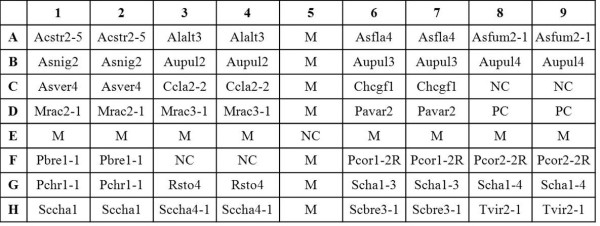
**Layout of oligonucleotide probes on the array (0.8 × 0.7 cm, 9 by 8 dots)**. The probe "PC" was a positive control and the probe "NC" was a negative control (tracking dye only). The probe "M", a position marker, was an irrelevant probe labeled with a digoxigenin molecule at the 5' end. The corresponding species names and sequences of all probes are listed in Table 2. All probes used for fungal identification were spotted on the array in duplicate.

The array (0.8 × 0.7 cm) contained 72 dots (9 by 8 dots), including 50 dots for species identification (duplicate dots of each of the 25 fungal-specific probes), 5 dots for negative control (probe code NC, tracking dye only), 2 dots for positive control (probe code PC), and 15 dots for the position marker (probe code M) (Figure 1). The oligonucleotide probes (10 μM) were drawn into wells of 96-well microtiter plates, and spotted onto positively charged nylon membrane (Roche, Mannheim, Germany) as described previously [28]. The layout of all probes on the array is shown in Figure 1.

### Array hybridization

The ITS region of a fungus was amplified by PCR using the forward primers (ITS1 and ITS5) and reverse primer (ITS4) as described in the previous section, with each primer being labelled with a digoxigenin molecule at the 5' end. The reagents and procedures for prehybridization, hybridization (55°C for 90 min), and color development using enzyme-conjugated anti-digoxigenin antibodies were previously described [28]. The hybridized spots (400 μm in diameter) could be read by the naked eye. A strain was identified as one of the species listed in Table 1 when both the positive control probe and the species-specific probe (or at least one of the multiple probes designed for a species) were hybridized (Table 2). The images of the hybridization pattern were captured by a scanner (PowerLook 3000; UMAX, Taipei, Taiwan).

### Isolation and identification of airborne fungi

Air samples were collected from three places (one hospital, one research laboratory, and one government office). The QuickTake 30 BioStage Pump kit (SKC Inc., Eighty Four, Pennsylvania, USA) was used to collect air samples. Fungal spores were collected on malt extract agar and Saubouraud dextrose agar for 3 min at a flow rate of 28.3 L/min. Spores trapped on agar plates were grown at 25°C for 3-7 days. Seventy colonies grown on agar plates were selected for identification by the array. The species names of the fungi identified by the array were further verified by sequencing of the ITS region and the D1/D2 domain of the large-subunit RNA gene; sequences in the two regions were found to be highly specific for a fungal species [29,30]. Species were identified by searching databases using the BLAST sequence analysis tool in the National Center for Biotechnology Information. If the result of array hybridization was in accordance with that of either ITS or D1/D2 domain sequencing, the identification made by the array was considered to be correct.

### Determination of detection limit

Detection limit was the lowest amount of fungal DNA that could be detected by the array. Serial 10-fold dilutions of DNAs of *Aspergillus fumigatus *BCRC 30502 and *A. versicolor *BCRC 31488 were used to determine the detection limits.

## Results

### Probe design

Initially, about 100 probes (data not shown) were designed to identify the 21 species listed in Table 1. Through extensive screening, many probes cross-reacted with heterologous species or produced weak hybridization signals with homologous species. Finally, 25 probes were selected for fabrication of the array (Table 2); these probes included 11 oligonculeotides published in our previous studies [19,26,30]. One or multiple probes were designed to identify a single species, depending on the availability of divergent sequences in the ITS region (Table 2). For most species, a single probe was enough to identify an individual microorganism. But one probe (code Chcgf1) was used to identify a group of three closely related species (*Chaeotomium cochlioides*, *C. globosum*, and *C. fumicola*) due to high interspecies similarities of the ITS sequences among these species. Conversely, some fungi displayed high intraspecies sequence divergence in the ITS regions and hence multiple probes were constructed to identify a single species. For example, two probes were used to identify each of the following species: *Mucor racemosus*, *Penicillium corylophilum*, *Scopulariopsis chartarum*, and *Stachybotrys chartarum*, and three probes were synthesized to identify *Aureobasidium pullulans *(Table 2).

### Sensitivity and specificity of the array

**Figure 2:**
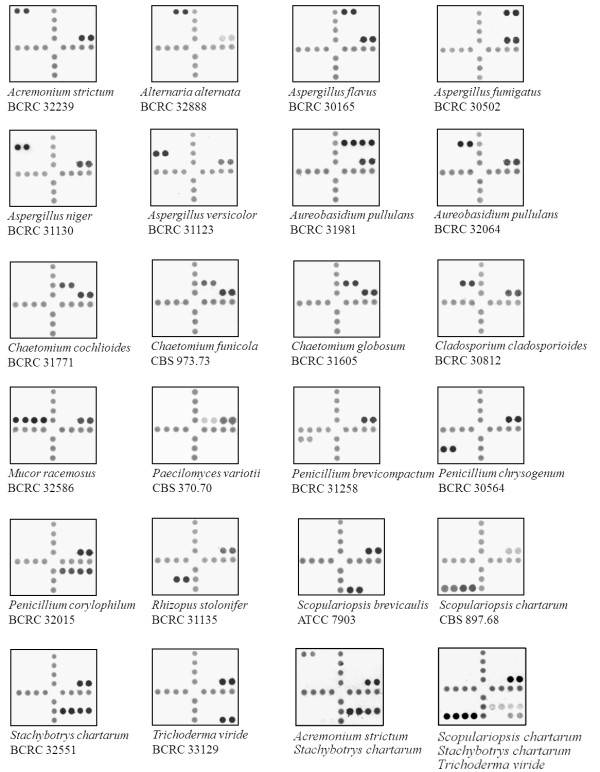
**Hybridization patterns of 21 species of fungi on the array**. The corresponding probes hybridized on the array are indicated in Figure 1, and the corresponding sequences of the hybridized probes are shown in Table 2. All probes used for fungal identification were spotted on the array in duplicate. The last two panels shows the simultaneous hybridization of two (*Acremonium strictum *and *Stachybotrys chartarum*) and three species (*Scopulariopsis chartarum*, *Stachybotrys chartarum*, and *Trichoderma viride*), respectively, on a single array.

A total of 139 reference strains, including 73 target and 66 nontarget strains, were analyzed by the array. The hybridization patterns of different fungal species are shown in Figure 2. Of the 73 target strains, 72 (98.6%) were correctly identified to the species or group level by the array, with one strain (*Trichoderma viride *BCRC 32054) being not identified (only the positive control was hybridized). Discrepancy analysis revealed that the strain BCRC 32054 had an ITS 1 and ITS 2 sequence similarity of 100% with *Trichoderma harzianum*, while the corresponding sequence similarities were only 78.7% and 87.2%, respectively, with a reference sequence of *Trichoderma viride *in GenBank (accessing no. X93978). It was obvious that *Trichoderma viride *BCRC 32054 was a misidentification of *Trichoderma harzianum*, a nontarget species in this study. Therefore, the sensitivity of the array was 100% (72/72). In addition, a collection of 66 nontarget strains (66 species) were used for specificity testing of the array (additional file 1). No cross-hybridization was observed for any strain analyzed and a specificity of 100% (66/66) was obtained.

### Detection limit of the array

Serial 10-fold dilutions of DNAs extracted from two strains (*Aspergillus fumigatus *BCRC 30502 and *A. versicolor *BCRC 31488) were used to determine the detection limits. For both strains, the detection limit of the array was 10 pg genomic DNA per assay; this amount of DNA was approximately equal to 270 cells (37 fg of DNA per cell of *Candida albicans*) [13].

### Identification of fungal strains isolated from air samples

The array was used to identify 70 fungal isolates recovered from the air samples in three buildings including one hospital (24 strains), one research laboratory (11 strains), and one office (35 strains). Among the 70 strains, 27 were identified to species level by the array, and 43 strains were not identified (nontarget species). The identified airborne fungi were *Aspergillus fumigatus *and *A. versicolor *(from a hospital), *A. niger *and *A. versicolor *(from a laboratory), and *Alternaria alternata*, *Aspergillus flavus*, *Cladosporium cladosporioides*, and *Penicillium chrysogenum *(from an office) (Table 3). Among the 27 strains identified by hybridization, 26 were correctly identified, as revealed by their morphological characteristics and sequencing of the ITS and ribosomal D1/D2 domains of the rRNA operons (Table 3). A strain (no. 12) was misidentified as *Acremonium strictum *by the array, since the ITS sequences demonstrated that the strain was *Acremonium implicatum*, a nontarget species. The remaining 43 non-identified strains from air samples belonged to nontarget species, as evidenced by their ITS and D1/D2 sequences (additional file 2). Some nontarget strains were only identified to the genus level by DNA sequencing since there were no corresponding ITS or D1/D2 sequence entries in the public database. Based on these results, the sensitivity and specificity of the array for identification of airborne fungi were 100% (26/26) and 97.7% (43/44), respectively. Among the 70 isolates recovered from air samples, 26 (37.1%) have potentials to cause allergy or adverse health problems in susceptible individuals.

**Table 3:** Identification of fungi isolated from indoor air by the array and by sequence analysis of the ITS and D1/D2 domain

Strain no.^*a*^	Species identification by			
	
	Array hybridization	ITS sequence (%)^a^	D1/D2 sequence (%)^a^	Best match
1	*Cladosporium cladosporioides*	*Cladosporium cladosporioides *(99.4)	*Cladosporium cladosporioides *(99.6)	*Cladosporium cladosporioides*
3	*Aspergillus versicolor*	*Aspergillus versicolor *(100)	*Aspergillus versicolor *(99.0)	*Aspergillus versicolor*
5	*Aspergillus niger*	*Aspergillus niger *(100)	*Aspergillus niger *(100)	*Aspergillus niger*
12	*Acremonium strictum*^*b*^	*Acremonium implicatum *(100)	*Acremonium *sp. (97.8)	*Acremonium implicatum*
13	*Cladosporium cladosporioides*	*Cladosporium cladosporioides *(100)	*Cladosporium cladosporioides *(100)	*Cladosporium cladosporioides*
28	*Alternaria alternata*	*Alternaria alternata *(100)	*Alternaria alternata *(98.6)	*Alternaria alternata*
33	*Aureobasidium pullulans*	*Aureobasidium pullulans *(100)	*Aureobasidium pullulans *(98.2)	*Aureobasidium pullulans*
35	*Aspergillus flavus*	*Aspergillus flavus *(100)	*Aspergillus flavus *(100)	*Aspergillus flavus*
38	*Aspergillus flavus*	*Aspergillus flavus *(99.8)	*Aspergillus flavus *(99.8)	*Aspergillus flavus*
41	*Cladosporium cladosporioides*	*Cladosporium cladosporioides *(100)	*Cladosporium cladosporioides *(99.6)	*Cladosporium cladosporioides*
42	*Cladosporium cladosporioides*	*Cladosporium cladosporioides *(99.8)	*Cladosporium cladosporioides *(99.8)	*Cladosporium cladosporioides*

43	*Cladosporium cladosporioides*	*Cladosporium cladosporioides *(100)	*Cladosporium cladosporioides *(99.8)	*Cladosporium cladosporioides*
44	*Cladosporium cladosporioides*	*Cladosporium cladosporioides *(100)	*Cladosporium cladosporioides *(100)	*Cladosporium cladosporioides*
45	*Cladosporium cladosporioides*	*Cladosporium cladosporioides *(100)	*Cladosporium cladosporioides *(99.6)	*Cladosporium cladosporioides*
47	*Cladosporium cladosporioides*	*Cladosporium cladosporioides *(100)	*Cladosporium cladosporioides *(99.6)	*Cladosporium cladosporioides*
48	*Cladosporium cladosporioides*	*Cladosporium cladosporioides *(100)	*Cladosporium cladosporioides *(99.6)	*Cladosporium cladosporioides*
49	*Aspergillus versicolor*	*Aspergillus versicolor *(99.8)	*Aspergillus versicolor *(99.3)	*Aspergillus versicolor*
50	*Aspergillus versicolor*	*Aspergillus versicolor *(100)	*Aspergillus versicolor *(99.3)	*Aspergillus versicolor*
52	*Aspergillus versicolor*	*Aspergillus versicolor *(99.8)	*Aspergillus versicolor *(99.1)	*Aspergillus versicolor*
55	*Aspergillus versicolor*	*Aspergillus versicolor *(100)	*Aspergillus versicolor *(99.8)	*Aspergillus versicolor*
56	*Aspergillus versicolor*	*Aspergillus versicolor *(100)	*Aspergillus versicolor *(98.6)	*Aspergillus versicolor*
58	*Aspergillus versicolor*	*Aspergillus versicolor *(100)	*Aspergillus versicolor *(99.1)	*Aspergillus versicolor*
59	*Aspergillus versicolor*	*Aspergillus versicolor *(100)	*Aspergillus versicolor *(99.3)	*Aspergillus versicolor*
60	*Aspergillus versicolor*	*Aspergillus versicolor *(100)	*Aspergillus versicolor *(99.1)	*Aspergillus versicolor*

64	*Aspergillus niger*	*Aspergillus niger *(99.8)	*Aspergillus niger *(99.1)	*Aspergillus niger*
67	*Aspergillus niger*	*Aspergillus niger *(99.8)	*Aspergillus niger *(99.6)	*Aspergillus niger*
68	*Aspergillus niger*	*Aspergillus niger *(99.8)	*Aspergillus niger *(100)	*Aspergillus niger*

^a^Values in parentheses are percentages of sequence similarities of the test isolates with the best-scoring sequences in the GenBank database.

^b^The strain was misidentified by array hybridization.

## Discussion

In this study, an oligonucleotide array was developed to identify 21 species of airborne fungi that are of health concern (Table 1). High sensitivity and specificity of the array were demonstrated by testing a collection of 138 reference strains and 70 isolates from air samples. Comparing with glass chip, the current membrane array is relatively simple, time-saving, and the test cost was quite low. In addition, only minimal instrumentation (a shaker and an incubator) is required for hybridization. The whole procedure for fungal detection by the array can be finished within a working day (8 h), starting from isolated colonies. The prominent feature of the current method is the use of a standardized protocol encompassing DNA extraction, ITS amplification, and membrane hybridization. The examinations of fungal reproductive structures, which are essential for classical identification, are not required by the present method.

In this study, one or multiple probes were designed to identify a single species, depending on the availability of divergent sequences in the ITS region (Table 2). The advantage of using multiple probes is the increased coverage of different strains of a species, but the disadvantage is the potential decrease of specificity due to the unpredictable cross-hybridizations caused by other irrelevant fungal species. The *T*_*m *_(melting temperature) values of probes used in this study ranged from 52.6 to 66.2°C, with some probes had *T*_*m *_lower than the hybridization temperature (55°C) (Table 2). However, clear signals were obtained for all target strains tested (Figure 2). Volokhov et al. [31] also reported the successful use of probes having *T*_*m *_values lower than the hybridization temperature for bacterial identification. The addition of several thymine bases to the end of a probe (Table 2) had the benefit of reducing steric hindrance between target DNA and the probe immobilized on a solid support [27]

In a previous study, an array targeting the 18S rRNA gene was developed to identify airborne fungi [24]. Since the 18S rRNA genes are highly conserved in closely related species, therefore species identification was based on hybridization patterns involving a combination of multiple probes rather than on species-specific probes [24]. The advantage of the current study is that all species were discretely identified by specific probes and the reading of results is very straightforward. The successful design of different probes was based on the known ITS sequences (Table 2), and multiple sequence alignment (interspecies and intraspecies) played an important role in finding out the regions that could be utilized for probe design. The present array is a powerful tool for the identification of important airborne fungi that may cause health problems in susceptible individuals. The array has the potential to be continually extended by including more probes, without significant increase of the cost or complexity. The current method permits a shorter time to achieve results as well as the correct identification of morphologically indistinguishable species.

Furthermore, the array was able to identify multiple fungal species at the same time as demonstrated in Figure 2 (the last two arrays). The DNAs of colonies from two (*Acremonium strictum *and *Stachybotrys chartarum*) or three species (*Scopulariopsis chartarum*, *Stachybotrys chartarum*, and *Trichoderma viride*) on agar plates were extracted in a tube, amplified by PCR, and hybridized to an array. All individual species were simultaneously identified. Furthermore, we also tried to directly detect fungi by trapping airborne spores in a buffer, followed by centrifugation, DNA extraction, and array hybridization. However, comparing with culture, the direct method was less sensitive and this might be due to the limited numbers of spores collected (data not shown). It is anticipated that by improving the sampling method, DNA extraction efficiency, and using nested PCR [26], the current array may have a potential to directly detect airborne fungi without an initial cultivation step. A possible method would be that PCR can be directly performed on the collected fungal spores, omitting the DNA extraction step that may lead to a significant loss of DNA for a very small sample. However, further investigation is needed to verify this hypothesis.

## Conclusions

Identification of airborne fungi by the array is highly reliable and accurate. The method can be used as an effective alternative to the conventional identification methods. The current array can greatly contribute to decipher the relationship between airborne fungi and adverse health effects.

## Competing interests

The authors declare that they have no competing interests.

## Authors' contributions

WH coordinated the project. SS and LS performed data analysis, specimen collection and testing. TCC contributed to study design, coordinated the experiment, and drafted the manuscript. All authors read and approved the final manuscript.

## Pre-publication history

The pre-publication history for this paper can be accessed here:

http://www.biomedcentral.com/1471-2334/11/91/prepub

## Supplementary Material

Additional file 1 **Additional Table 1: Nontarget fungi used for specificity testing of the array**. 66 strains of nontarget fungi used to test the specificity of the array developed in this study Click here for file

Additional file 2 **Additional Table 2: Fungal strains not identified by the array and identification of these strains by sequencing of the ITS and D1/D2 domain**. Identification of 44 air fungal isolates not identified by the array by sequencing of the ITS and D1/D2 domain Click here for file
