# Supplementary material for: Rapid identification of allergenic and pathogenic molds in environmental air by an oligonucleotide array
Source: BMC Infect Dis. 2011 Apr 13;11:91. doi: 10.1186/1471-2334-11-91 (PMC3100263; doi:10.1186/1471-2334-11-91)
Supplement: Additional file 1 — Additional Table 1: Nontarget fungi used for specificity testing of the array. 66 strains of nontarget fungi used to test the specificity of the array developed in this study [file 1471-2334-11-91-S1.PDF]

Additional Table 1. Nontarget fungi used for specificity testing of the array

Species	Strain no.
Absidia coerulea	BCRC 30897
Absidia corymbifera	CBS 101.51
Absidia fusca	BCRC 33045
Acremonium chrysogenum	BCRC 31697
Acremonium falciforme	CBS 475.67
Acremonium thermophilum	BCRC 32323
Amylomyces rouxii	BCRC 32233
Apophysomyces elegans	CBS 658.93
Arthroderma borellii	CBS 967.68
Arthroderma uncinatum	CBS 180.64
Aspergillus carbonarius	BCRC 30001
Aspergillus clavatus	BCRC 31116
Aspergillus japonicus	BCRC 30022
Aspergillus nidulans	BCRC 30100
Aspergillus sulphureus	BCRC 30139
Aspergillus terreus	BCRC 30135
Beauveria bassiana	BCRC 32840[T]
Bipolaris hawaiiensis	CBS 103.97
Bipolaris spicifera	CBS 274.52
Blastomyces dermatitidis	ATCC 24327
Cladophialophora carrionii	CBS 410.96
Cladosporium herbarum	BCRC 33511
Chaetomium madrasense	BCRC 31608
Cokeromyces recurvatus	CBS 168.59
Cunninghamella bertholletiae	CBS 182.84
Curvularia geniculata	CBS 386.69
Curvularia verruculosa	CBS 147.63
Exophiala nidulans	BCRC 32208
Exophiala werneckii	ATCC 36317[T]
Fonsecaea compacta	CBS 212.77
Fusarium moniliform	BCRC 31492
Fusarium oxysporum	CBS 798.95
Fusarium verticillioides	CBS 539.79
Geotrichum klebahnii	CBS 179.30
Madurella grisea	ATCC 200278
Madurella mycetomatis	CBS 201.38

Malbranchea filamentosa	ATCC 48174[T]
Microsporum audouinii	ATCC 10008
Microsporum cookei	CBS 129.67
Microsporum duboisii	CBS 349.49
Microsporum ferrugineum	ATCC 11992
Microsporum gallinae	CBS 221.55
Microsporum praecox	CBS 288.55
Mucor flavus	CBS 673.66
Paecilomyces javanicus	BCRC 35511
Paecilomyces lilacinus	BCRC 31616
Paecilomyces marquandii	CBS 106.85
Paecilomyces viridis	CBS 348.65[T]
Paracoccidioides brasiliensis	ATCC 32069
Penicillium lividum	BCRC 31673
Penicillium marneffei	CBS 334.59[T]
Phialophora verrucosa	ATCC 4806
Pichia anomala	BCRC 22583
Rhinocladiella aquaspersa	CBS 313.73
Rhizomucor pusillus	BCRC 33122[T]
Rhizopus azygosporus	BCRC 31158
Rhizopus homothallicus	BCRC 31146
Rhizopus oryzae	BCRC 31145
Scedosporium prolificans	CBS 100390
Scopulariopsis acremonium	IHEM 6569
Scopulariopsis fusca	CBS 872.68
Sporothrix schenckii var. *schenckii*	CBS 345.53
Trichophyton terrestre	CBS 464.62
Trichosporon asahii	CBS 2479
Trichosporon inkin	BCRC 21503
Ulocladium conscortiale	CBS 104.31
Total no. of strains	66

[a]ATCC, American Type Culture Collection, Manassas, Virginia, USA; BCRC, Bioresources Collection and Research Center, Hsinchu, Taiwan, Republic of China; CBS, Centraalbureau voor Schimmelcultures, Utrecht, The Netherlands.

[T]Type strain.
