# Supplementary material for: Rapid identification of allergenic and pathogenic molds in environmental air by an oligonucleotide array
Source: BMC Infect Dis. 2011 Apr 13;11:91. doi: 10.1186/1471-2334-11-91 (PMC3100263; doi:10.1186/1471-2334-11-91)
Supplement: Additional file 2 — Additional Table 2: Fungal strains not identified by the array and identification of these strains by sequencing of the ITS and D1/D2 domain. Identification of 44 air fungal isolates not identified by the array by sequencing of the ITS and D1/D2 domain [file 1471-2334-11-91-S2.PDF]

Additional Table 2. Fungal strains not identified by the array and identification of these strains by sequencing of the ITS and D1/D2 domain

Strain no.[a]	Species identification by			Best match
	Array hybridization	ITS sequencing (%)[b]	D1/D2 sequence (%)[b]	
2	NI[a]	*Fusarium* sp. (100)	*Fusarium* sp. (99.8)	*Fusarium* sp.
4	NI	*Eurotiomycete* sp. (99.4)	*Penicillium marneffei* (98.7)	*Eurotiomycete* sp.
6	NI	*Penicillium pinophilum* (100)	*Penicillium pinophilum* (99.6)	*Penicillium pinophilum*
7	NI	*Penicillium oxalicum* (100)	*Penicillium oxalicum* (100)	*Penicillium oxalicum*
8	NI	*Penicillium oxalicum* (100)	*Penicillium oxalicum* (100)	*Penicillium oxalicum*
9	NI	*Microsphaeropsis arundinis* (100)	*Microsphaeropsis arundinis* (100)	*Microsphaeropsis arundinis*
10	NI	*Aspergillus aculeatus* (100)	*Aspergillus aculeatus* (100)	*Aspergillus aculeatus*
11	NI	*Microsphaeropsis arundinis* (100)	*Microsphaeropsis arundinis* (100)	*Microsphaeropsis arundinis*
14	NI	*Acremonium implicatum* (99.8)	*Acremonium implicatum* (99.8)	*Acremonium implicatum*
15	NI	*Penicillium* sp. (98.8)	*Penicillium* sp. (99.8)	*Penicillium* sp.
16	NI	*Fusarium* sp. (100)	*Fusarium* sp. (99.8)	*Fusarium* sp.
17	NI	*Penicillium oxalicum* (100)	*Penicillium oxalicum* (99.5)	*Penicillium oxalicum*
18	NI	*Aspergillus aculeatus* (100)	*Aspergillus aculeatus* (100)	*Aspergillus aculeatus*
19	NI	*Neurospora intermedia* (100)	*Neurospora intermedia* (99.6)	*Neurospora intermedia*
20	NI	*Neurospora intermedia* (100)	*Neurospora intermedia* (99.6)	*Neurospora intermedia*
21	NI	*Penicillium sclerotiorum* (100)	*Penicillium sclerotiorum* (98.2)	*Penicillium sclerotiorum*
22	NI	*Aspergillus aculeatus* (100)	*Aspergillus aculeatus* (100)	*Aspergillus aculeatus*
23	NI	*Fusarium* sp. (99.1)	*Fusarium* sp. (99.6)	*Fusarium* sp.
24	NI	*Neurospora intermedia* (100)	*Neurospora intermedia* (99.6)	*Neurospora intermedia*
25	NI	*Penicillium citrinum* (100)	*Penicillium citrinum* (100)	*Penicillium citrinum*

26	NI	*Penicillium herquei* (100)	*Penicillium herquei* (100)	*Penicillium herquei*
27	NI	*Gibberella moniliformis* (99.8)	*Gibberella moniliformis* (99.6)	*Gibberella moniliformis*
29	NI	*Aspergillus aculeatus* (100)	*Aspergillus aculeatus* (99.8)	*Aspergillus aculeatus*
30	NI	*Fusarium* sp. (100)	*Fusarium* sp. (99.8)	*Fusarium* sp.
31	NI	*Penicillium citrinum* (100)	*Penicillium citrinum* (99.5)	*Penicillium citrinum*
32	NI	*Penicillium oxalicum* (100)	*Penicillium oxalicum* (99.8)	*Penicillium oxalicum*
34	NI	*Fusarium* sp. (100)	*Fusarium* sp. (99.8)	*Fusarium* sp.
36	NI	*Arthrinium euphorbiae* (98.0)	*Arthrinium sacchari* (98.9)	*Arthrinium* sp.
37	NI	*Montagnulaceae* sp. (98.7)	*Leptosphaerulina chartarum* (98.8)	Not identified
39	NI	*Epicoccum nigrum* (100)	*Epicoccum nigrum* (99.1)	*Epicoccum nigrum*
40	NI	*Fusarium* sp. (100)	*Fusarium* sp. (99.8)	*Fusarium* sp.
46	NI	*Aspergillus aculeatus* (99.6)	*Aspergillus aculeatus* (100)	*Aspergillus aculeatus*
51	NI	*Aspergillus unguis* (99.8)	*Aspergillus unguis* (100)	*Aspergillus unguis*
53	NI	*Cladosporium sphaerospermum* (100)	*Cladosporium sphaerospermum* (99.8)	*C. sphaerospermum*
54	NI	*Aspergillus unguis* (100)	*Aspergillus unguis* (100)	*Aspergillus unguis*
57	NI	*Aspergillus aculeatus* (100)	*Aspergillus aculeatus* (99.7)	*Aspergillus aculeatus*
61	NI	*Aspergillus aculeatus* (100)	*Aspergillus aculeatus* (99.5)	*Aspergillus aculeatus*
62	NI	*Penicillium purpurogenum* (99.2)	*Penicillium purpurogenum* (99.8)	*Penicillium purpurogenum*
63	NI	*Aspergillus aculeatus* (99.3)	*Aspergillus aculeatus* (100)	*Aspergillus aculeatus*
65	NI	*Cochliobolus* sp. (99.0)	*Cochliobolus lunatus* (99.8)	*Cochliobolus lunatus*
66	NI	*Dothideomycete* sp. (99.8)	*Cochliobolus lunatus* (99.5)	*Cochliobolus lunatus*
69	NI	*Cladosporium sphaerospermum* (99.3)	*Cladosporium sphaerospermum* (99.5)	*C. sphaerospermum*
70	NI	*Cochliobolus lunatus* (99.6)	*Cochliobolus lunatus* (99.5)	*Cochliobolus lunatus*

[a]Not identified by the array.

[b]Values in parentheses are percentages of ITS sequence similarities of the test isolates with the best-scoring sequences in the database of GenBank.
